# A Combined Experimental-Numerical Framework for Assessing the Load-Bearing Capacity of Existing PC Bridge Decks Accounting for Corrosion of Prestressing Strands

**DOI:** 10.3390/ma14174914

**Published:** 2021-08-29

**Authors:** Dario De Domenico, Davide Messina, Antonino Recupero

**Affiliations:** Department of Engineering, University of Messina, Villaggio S. Agata, 98166 Messina, Italy; davide.messina@unime.it (D.M.); antonino.recupero@unime.it (A.R.)

**Keywords:** bridges, prestressed concrete bridge decks, operational modal analysis, dynamic identification, corrosion, corroded strands, structural vulnerability assessment, structural health monitoring, nonlinear static analysis

## Abstract

Bridges constitute important elements of the transportation network. A vast part of the Italian existing infrastructural system dates to around 60 years ago, which implies that the related bridge structures were constructed according to past design guidelines and underwent a probable state of material deterioration (e.g., steel corrosion, concrete degradation), especially in those cases in which proper maintenance plans have not been periodically performed over the structural lifetime. Consequently, elaborating rapid yet effective safety assessment strategies for existing bridge structures represents a topical research line. This contribution presents a systematic experimental-numerical approach for assessing the load-bearing capacity of existing prestressed concrete (PC) bridge decks. This methodology is applied to the Longano PC viaduct (southern Italy) as a case study. Initially, natural frequencies and mode shapes of the bridge deck are experimentally identified from vibration data collected in situ through Operational Modal Analysis (OMA), based on which a numerical finite element (FE) model is developed and calibrated. In situ static load tests are then carried out to investigate the static deflections under maximum allowed serviceability loads, which are compared to values provided by the FE model for further validation. Since prestressing strands appear corroded in some portions of the main girders, numerical static nonlinear analysis with a concentrated plasticity approach is finally conducted to quantify the effects of various corrosion scenarios on the resulting load-bearing capacity of the bridge at ultimate limit states. The proposed methodology, encompassing both serviceability and ultimate conditions, can be used to identify critical parts of a large infrastructure network prior to performing widespread and expensive material test campaigns, to gain preliminary insight on the structural health of existing bridges and to plan a priority list of possible repairing actions in a reasonable, safe, and costly effective manner.

## 1. Introduction

Most of the existing bridges and viaducts in Italy were constructed around 60 years ago, thus complying with past design guidelines. Many of these bridges were realized with prestressed concrete (PC) deck (with either pretensioned strands or post-tensioned tendons) [1,2] and reinforced concrete (RC) piers. In addition to the differences between design regulations in force at the time of bridge construction and the current design standards, the vulnerability of these structures is further increased by material deterioration phenomena occurred over the structural lifetime, primarily corrosion of steel element (both reinforcement bars of RC piers and prestressing strands of PC main girders). Durability issues of these structures, which are related not only to construction details, but also (and most importantly) to the frequency and extensiveness of inspections and related maintenance interventions, have been often neglected and not considered as key performance objectives in the past few decades. However, recent collapses of bridges occurred in Italy in recent years have revealed the importance of such aspects for the risk mitigation of existing bridges and for the safety of the overall infrastructure network [3]. A representative example is given by the Polcevera viaduct failure in Genoa [4] occurred on 14 August 2018, causing social and economic losses estimated in 43 deaths and around 2 years of downtime. Consequently, the managing bodies of the road network (be them national agencies or private companies) are currently performing a comprehensive survey of the bridge structures through experimental measurements accompanied by numerical investigations aimed at safety assessment of existing bridges. The aim is to rapidly identify potential critical portions of the road network without performing extensive material tests, based on which specific plans of retrofitting actions and, above all, intervention priorities can be adopted in a timely and precise manner [5]. Retrofitting interventions to extend bridge service life can be either localized to specific, weak zones, such as applying composite strips to bridge deck slabs [6], or designed to control the overall bridge structural behavior, such as via the introduction of passive energy dissipation systems (e.g., viscous dampers) when an insufficient seismic capacity is detected [7].

Until one year ago, inspection activities on Italian bridges were regulated by guidelines established in a Technical Circular issued by the Italian Ministry of Public Works and dating back to 1967 [8]. The increasing importance of safety assessment of existing bridges has led to the development of a new document entitled “Guidelines for classification and risk management, safety assessment, and structural health monitoring of existing bridges” [9], approved in April 2020 and compliant with the Italian Technical Rules for Constructions [10] (in sequel simply referred to as NTC2018) in terms of load combinations, safety checks, load models and partial safety factors for the structural analysis of bridge structures. This document has allowed an extensive classification of the entire existing bridge stock, based on a simplified risk analysis combined with a multi-hazard methodology (including structural, seismic, geotechnical, and hydraulic hazards) to define an overall attention class of the bridge.

One of the most critical structural systems identified in the above-mentioned guidelines [9] and in recent inspections of existing bridges concerns PC girders that may be vulnerable to a series of structural issues, such as construction defects in the anchorage zones, prestressing steel relaxation losses [11], durability problems and deterioration phenomena, primarily corrosion on steel tendons [12]. Indeed, RC and PC structures placed in aggressive environments (e.g., featured by high concentration of sulphates and/or chlorides from marine environments, deicing salts, etc.) suffer from corrosion problems related to carbonation of concrete and electrochemical oxidization of steel tendons. Apart from the reduction of the steel area, the iron oxides and rust, occupying a volume six times higher than the original metal, cause transversal tensile stresses in steel [13], thus leading to diffuse microcracking in the surrounding concrete and resulting in spalling of concrete cover, deterioration of the bond links between concrete and steel [14,15], reduction of the sectional capacity [16] and consequent modification of the structural behavior [17]. These effects are more impactful on the load-bearing capacity of PC structures than ordinary RC structures, because steel strands operate at higher levels of stress (up to five times) than those of mild steel re-bars. Consequently, evaluating to what extent corrosion of steel strands (in pretensioned PC girders) or steel tendons (in post-tensioned PC girders) affects the load-bearing capacity of the bridge structure is of utmost importance.

The load-carrying capacity assessment of existing bridge structures represents a topical research line, as confirmed by the wide number of experimental [18], numerical [19], and combined experimental-numerical works [20] in the literature, including multi-level approaches [21] and probabilistic, time-dependent methods [22,23] accounting for degradation of material parameters, especially corrosion of steel. Generally, numerical analysis is assisted by field test results such as diagnostic load tests [24,25], or other non-destructive testing techniques like impact echo [26].

This contribution falls into this research line and proposes a rapid yet effective approach for the assessment of the safety conditions of existing bridge structures under both serviceability and ultimate loading conditions. In particular, the focus of the work is on PC bridge decks representing a common structural scheme for medium span bridges. The proposed methodology consists in a combined experimental-numerical framework that is applied to the case study of the PC bridge deck of the Longano viaduct, Barcellona P.G., Italy, and whose main steps are summarized as follows. First, dynamic identification of natural frequencies and mode shapes of the bridge deck is performed through operational modal analysis (OMA) based on vibration data collected in situ. These measurements are subsequently used for calibrating a numerical FE model of the bridge deck using 1D beam elements for girders and transverse diaphragms. Experimental static load tests on the bridge deck are then carried out in situ under increasing loads levels (in three subsequent phases) up to the maximum allowed serviceability loads, and measuring the corresponding deflections associated with each load step. The static tests performed in situ are also simulated through the previously calibrated FE model, by applying appropriate tributary loads on the beam elements and comparing the obtained numerical deflections with those measured experimentally for further validation. Once the FE model is calibrated and validated, additional numerical analyses are performed to investigate the bridge behavior at ultimate limit states. Macroscopic observations on the bridge deck reveal a state of advanced corrosion in some prestressing steel strands, which may significantly reduce the cross-sectional resisting capacity and may seriously affect the ultimate structural behavior of the bridge deck. To this aim, static nonlinear analysis with a concentrated plasticity approach is finally performed to assess the influence of various corrosion scenarios of the prestressing strands on the resulting load-bearing capacity of the bridge deck. The proposed experimental-numerical framework makes it possible to preliminarily assess the structural behavior of the bridge under simultaneous service and ultimate load conditions prior to performing extensive material test campaigns, unlike other methods from the literature. This preliminary assessment is a crucial goal to plan appropriate retrofitting interventions in a large infrastructure network. Moreover, the presented methodology enables one to investigate the influence of various corrosion scenarios of prestressing strands under extreme loading conditions. The latter information can be extremely useful to evaluate, in an effective and rapid manner, the structural vulnerability of bridge structures in those circumstances in which experimental measures of the actual strand corrosion rate are available, or when imminent inspections are designed to investigate material degradation phenomena in situ.

## 2. Description of the Longano Viaduct

**Figure 1:**
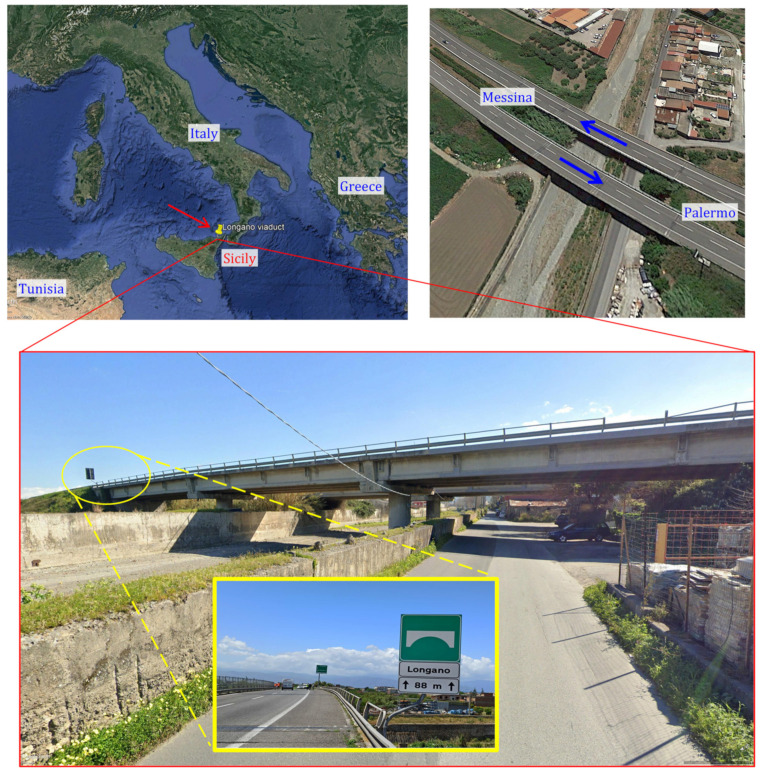
Location and photograph of the Longano viaduct, Barcellona P.G., Italy analyzed in this paper as a case study. (Base map © 2021 Basarsoft US Department of State Geographer Data, U.S. Navy, GEBCO Image Landsat/Copernicus).

The Longano viaduct was built in 1970. It is located in the municipality of Barcellona Pozzo di Gotto, Sicily (southern Italy), approximately 1 km in front of the Tyrrhenian Sea. This viaduct belongs to the A20 infrastructure network connecting the provinces of Messina and Palermo; location and photograph of the viaduct are provided in Figure 1.

**Figure 2:**
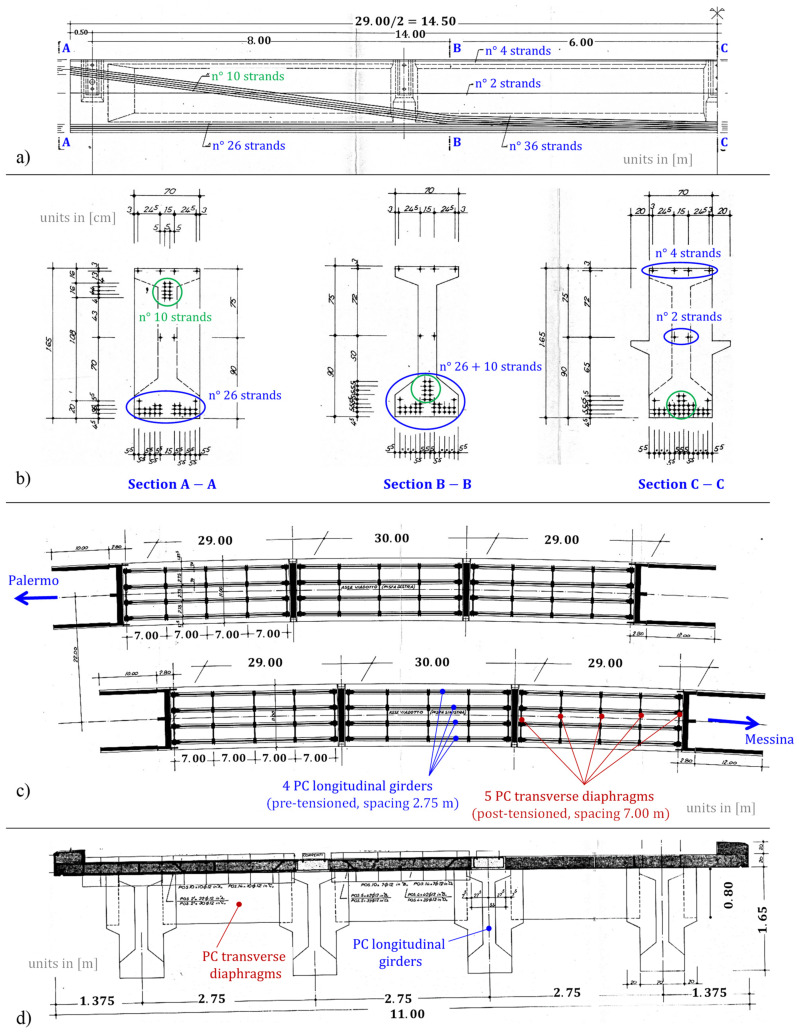
Original design drawings of the Longano viaduct: (**a**) configuration of the prestressing strands in longitudinal girders; (**b**) cross-sections of PC girders; (**c**) horizontal section of the bridge deck (two independent roadways); (**d**) transverse section of the bridge deck.

With an overall length of 88 m, the viaduct is articulated in two twin parts, each representing an independent roadway, with a straight longitudinal configuration and modest altimetric variations. Each roadway (one following the traffic direction from Messina to Palermo and the other in the opposite direction) is composed of: 2 circular piers of heights 5.50 m and 6.00 m, having diameter 2.20 and equipped with spiral reinforcement and Dywidag prestressing steel bars with diameter 32 mm; 2 abutments with height 6 m and 7 m in RC; 3 spans with a statically determinate scheme in PC, having length equal to 29 m (two external spans) and 30 m (central span); RC slab having thickness 20 cm and width 11 m, including curbs and parapets. The structural scheme is composed of an assembly of 4 longitudinal I-shaped PC girders, spaced 2.75 m, with 42 pretensioned steel 0.6″ strands, of which 26 in the bottom flange, 2 in the web, 4 in the top flange (all of them arranged in straight configuration) and the remaining 10 inclined from the upper portion of the section (near the girder supports) to the bottom flange via intermediate deviators located 8 m from the supports (see Figure 2a,b) that show just one half of the girder reinforcement configuration up to midspan, the other half being perfectly symmetric). The longitudinal I-shaped PC girders, of height 1.65 m and width 0.70 m, are mutually connected through transverse PC (post-tensioned) diaphragms (5 per each span), having rectangular section (20 cm × 80 cm) and spaced 7.00 m (see Figure 2c,d) showing the horizontal and transverse sections of the bridge deck). The PC girders are simply supported on neoprene bearings having dimensions 0.60 m × 0.45 m × 0.064 m. In the transverse direction, the bridge deck has a couple of RC seismic restraints realized in the pier cap and in the abutments. As to the materials adopted, the original design drawings report concrete grade C35/45 (cylindrical characteristic strength ≥35 MPa), prestressing strands with ultimate tensile strength greater than 1667 MPa and reinforcement bars (of RC slab) with yielding stress greater than 430 MPa and ultimate strength greater than 540 MPa.

## 3. Visual Inspection and Motivations of the Experimental Campaign

Considering the location of the Longano viaduct (just 1 km from the sea) and the fact that the bridge was built more than 50 years ago, some deterioration phenomena might have occurred in the materials, mainly ascribed to aggressive agents like chlorides from the marine environment. These hypothetical considerations are supported by the bridge deterioration state actually detected by visual inspection, whose outcomes are briefly illustrated here and form the basis of the experimental campaign planned afterwards.

**Figure 3:**
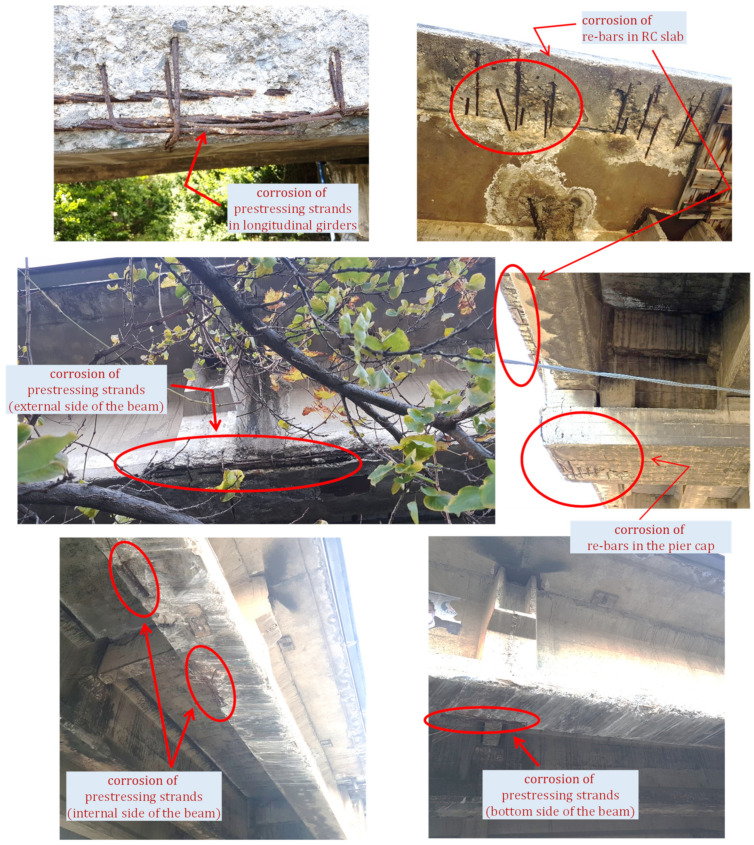
Widespread corrosion state of the Longano viaduct detected from visual inspection.

According to the multi-level approach recommended by the Italian Guidelines for bridges [9], after the level-0 process of data collection of the bridge, including location, design drawings to identify structural system, check for past reports of retrofit interventions, etc. [27], the next level 1 concerns a visual inspection on the bridge with the aim to detect existing damage signs, including (among others) cracking spots, spalling of concrete cover and, most importantly, steel corrosion. A set of photographs taken on different parts of the Longano viaduct is reported in Figure 3. It is clearly noticed that there is a widespread deterioration state along the bridge deck with concrete cover completely removed in extensive portions of the girder soffit and marked corrosion phenomena in prestressing strands (more pronounced in the strands located in the bottom part of the I-shaped section) as well as in the mild steel bars of the RC slab. On the contrary, the inspection does not reveal any damage state in the transverse diaphragms (neither cracking, nor concrete spalling, nor corrosion of steel tendons).

Based on the preliminary data acquired during the visual inspection, it is reasonable to think that the observed corrosion phenomena of prestressing strands might have reduced the sectional capacity of the PC longitudinal girders. Depending on the initial prestressing force considered in the design stage and the extensiveness of the corrosion process (which are two unknown parameters), theoretically speaking, the reduced steel area of the strands may either affect the ultimate limit states only, or also influence the sectional capacity under serviceability loads. Indeed, it is not easy to foresee whether the corrosion rate is such to produce the cracking state under service loading conditions. Should this critical scenario of cracking under service loads occur, the evolution of damage state detected from the visual inspection might evolve more and more rapidly under service traffic loads. It is, therefore, of extreme importance to check the structural behavior of the bridge under serviceability loads (up to the maximum loads allowed by the Italian regulations NTC2018 [10]). To this aim, static load tests on the bridge deck are planned to reproduce such extreme service loading conditions corresponding to the characteristic combination of loads reported in the Eurocode 0 [28] §6.5.3, with unitary combination coefficients for variable actions (normally used for irreversible limit states). However, before performing such static load tests, it is decided to preliminarily identify natural frequencies, mode shapes and damping ratios of the bridge through OMA (collecting free vibration data). These dynamic characteristics serve as useful indicators of the bridge structural health and are, therefore, checked and compared prior to and after the static load tests to assess whether some irreversible damage is caused by the applied loads. The description of the in situ experimental campaign is reported in the next section.

## 4. In Situ Experimental Dynamic and Static Tests under Service Loads

### 4.1. Operational Modal Analysis and Identification of Dynamic Parameters

The aim of dynamic tests performed in this experimental campaign is to identify dynamic parameters (natural frequencies and mode shapes, as well as damping ratios) of the bridge deck through free vibration analysis. As already said, the knowledge of such dynamic parameters is strictly related to the geometric and mechanical properties of the Longano viaduct and is, therefore, useful for a rapid control of the bridge structural health in operating conditions. Modal identification is typically performed through either operational modal analysis (OMA) [29], which is an output-only technique in which the vibrations of the structure are measured without knowing the excitation characteristics, or experimental modal analysis (EMA) [30], which is an input-output technique measuring the structural response to a given (known) dynamic excitation (e.g., artificial exciter or vibrodine). In structural health monitoring, OMA is more widely used because of the ease of realization, low cost of execution, and possibility of exerting a relatively weak excitation, which avoids exceeding the linear elastic regime of the structure. This technique is used in this work to identify natural frequencies and mode shapes of the Longano viaduct.

**Figure 4:**
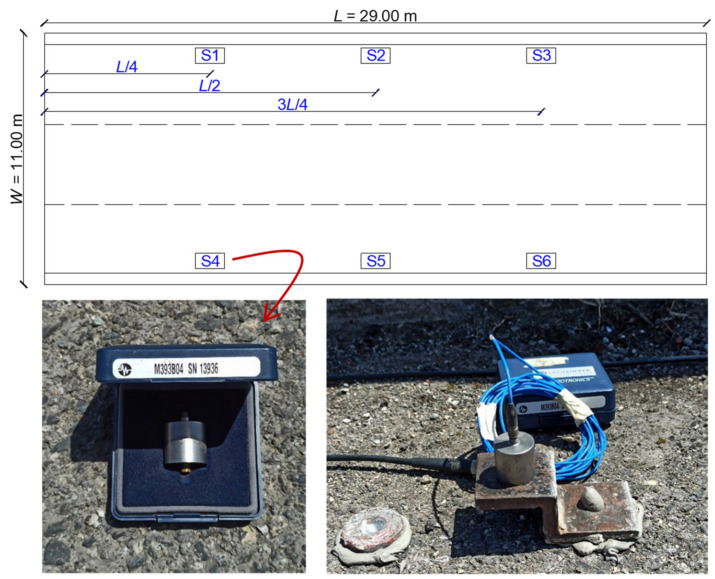
Position of the six accelerometers (S1–S6) on the bridge deck (**top**) and representative accelerometer photograph (**bottom**).

Six accelerometers with vertical axis are installed on the bridge deck (following an identical scheme in the two independent roadways). The accelerometers are placed symmetrically with respect to the roadway width and are rigidly anchored to the RC slab at 1/4, 1/2 and 3/4 of the span, cf. Figure 4. The signal acquisition is performed with sampling rate Δt=0.001 s (sampling frequency $f_{s}=1\;\mathrm{kHz}$). The range of accelerometer sensitivity is (912–1006) mV/g for the six devices employed, with g being the acceleration of gravity.

**Figure 5:**
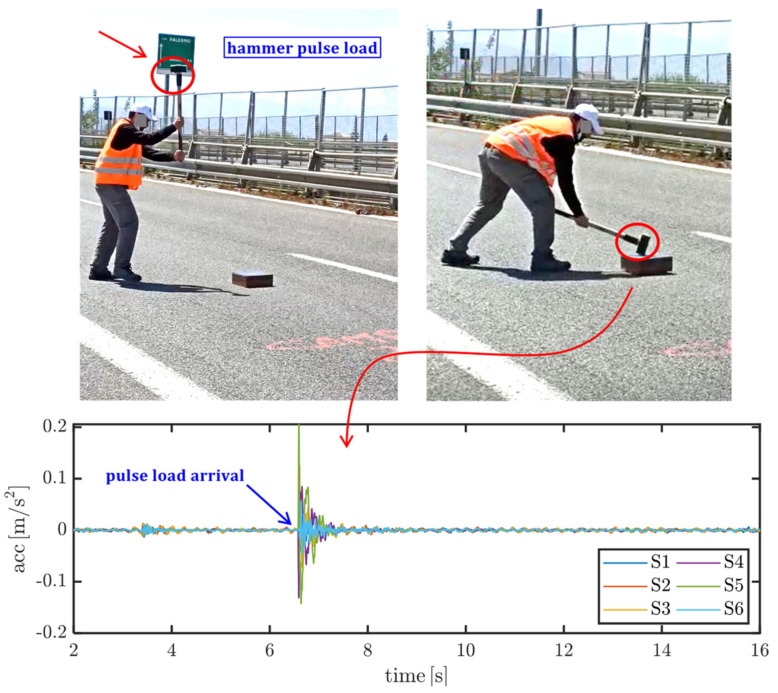
Dynamic excitation of the Longano viaduct with a pulse load produced by a 4 kg hammer blow and related filtered acceleration signals.

**Figure 6:**
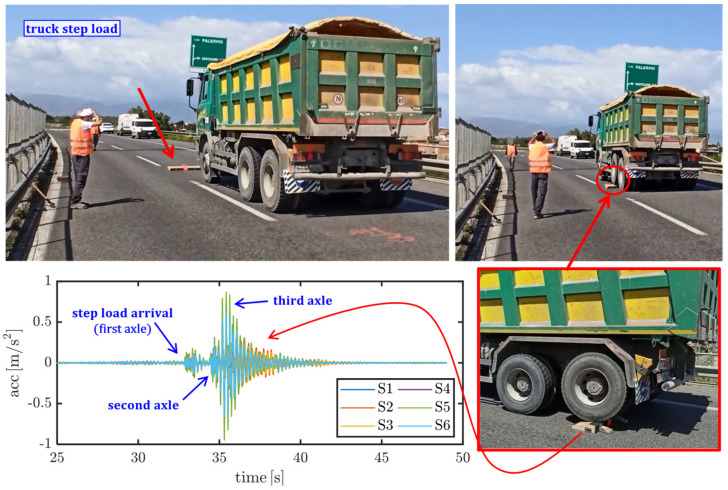
Dynamic excitation of the Longano viaduct realized with the transit of a three-axle 347 kN gross-weight truck on a 12 cm step and related filtered acceleration signals.

Two different excitations are considered to trigger free vibrations of the bridge deck and to identify the largest possible number of modal parameters: (1) pulse load exerted by a 4 kg hammer, as illustrated in Figure 5, which is applied to three different positions, namely at a quarter of the span (from either side) and at midspan; (2) step load exerted by the transit of a three-axle heavy truck (with gross weight of 347 kN) passing on a 12 cm step, as depicted in Figure 6, which is applied to three different positions, namely at a quarter of the span (from either side) and at midspan.

**Figure 7:**
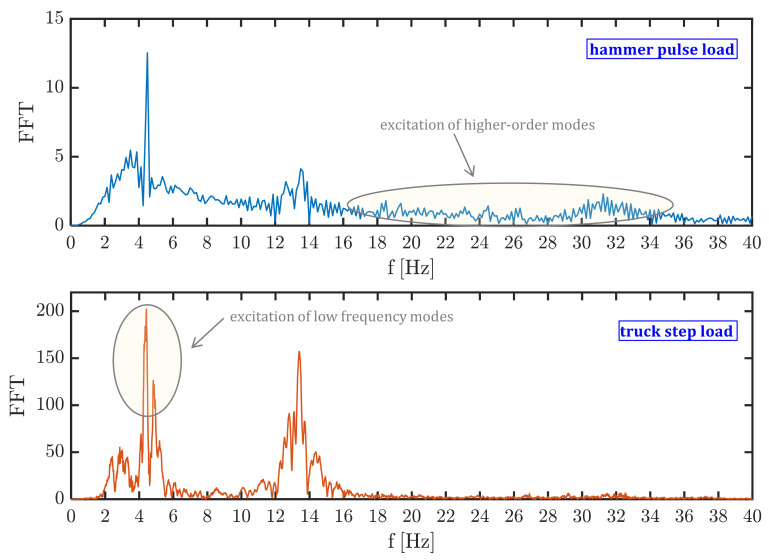
FFT of the S1 signal under hammer pulse load (**top**) and truck step load (**bottom**).

In the postprocessing phase of the recorded signals, the acceleration time histories are subjected to a Butterworth filter of order 6 to remove the frequencies falling outside the range of interest, here selected as (3–40) Hz. Subsequently, each recording signal is processed in the frequency domain through the fast Fourier transformation (FFT), a representative example of which is shown in Figure 7. It is worth noting that the two types of excitations (hammer pulse and truck transit) affect the modal response to a rather different extent: the hammer blow pulse generally amplifies a wider range of frequencies, including higher order modes, whereas the transit of the heavy truck mainly excites the first (low frequency) modes of vibration, cf. again Figure 7.

Due to the vicinity of the first frequencies to each other, the natural frequencies and mode shapes are identified through the frequency domain decomposition (FDD) technique [31]. The FDD is based on the calculation of the power spectral density (PSD) matrix of the output (i.e., of the structural response of the bridge deck $y\left(t\right)$) $G_{yy}\left(\omega \right)$ that, under the assumption of a white noise input (broad-banded excitation) and lightly damped system, is proportional to the frequency response function matrix and, consequently, is useful to identify dynamic parameters of the structure. According to the FDD, the spectral matrix is decomposed into a series of auto spectral density functions, each associated with a single degree of freedom (SDOF) system. The PSD matrix $G_{yy}$ provides information on how the signal power is distributed along the frequency spectrum; this matrix has three dimensions $\left[m\times m\times N_{f}\right]$, where m is the number of recorded signals (in this case m=6) and $N_{f}$ is the number of discrete output frequencies. This matrix contains the auto spectral density functions along the diagonal and the cross spectral density function as off diagonal terms and is here estimated through the built-in MATLAB function cpsd [32] from the filtered accelerometer signals. Once an estimate of the output PSD matrix ${\hat{G}}_{yy}$ is obtained at discrete frequencies $\omega_{i}$, the singular value decomposition (SVD) of this Hermitian matrix is performed as follows:

(1) $${\hat{G}}_{yy}\left(\omega_{i}\right)=U_{i}S_{i}U_{i}^{H},$$

where $U_{i}$ is the unitary (complex orthogonal) matrix in which each column collects the singular vectors, $S_{i}$ is a diagonal matrix collecting the scalar singular values, i.e., the eigenvalues of the matrix $G_{yy}\left(\omega \right)$ in descending order and $U_{i}^{H}$ is the conjugate transpose matrix of $U_{i}$. The SVD is performed through the built-in MATLAB function svd [32] from the estimated PSD matrix and provides six singular values and corresponding singular vectors.

**Figure 8:**
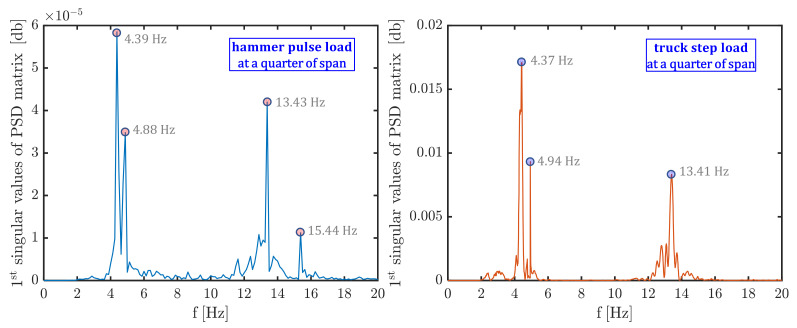
Identification of natural frequencies based on peak picking from the first singular values of the PSD matrix.

The determination of natural frequencies and mode shapes from the PSD matrix assumes that near the resonant frequency of the structure (where there is only one dominant mode) the singular vectors represent estimates of the mode shapes, and the corresponding singular values represent estimates of corresponding natural frequencies. Some representative examples of singular values of the PSD matrix and the resulting identification of the natural frequencies by peak picking technique is illustrated in Figure 8.

**Figure 9:**
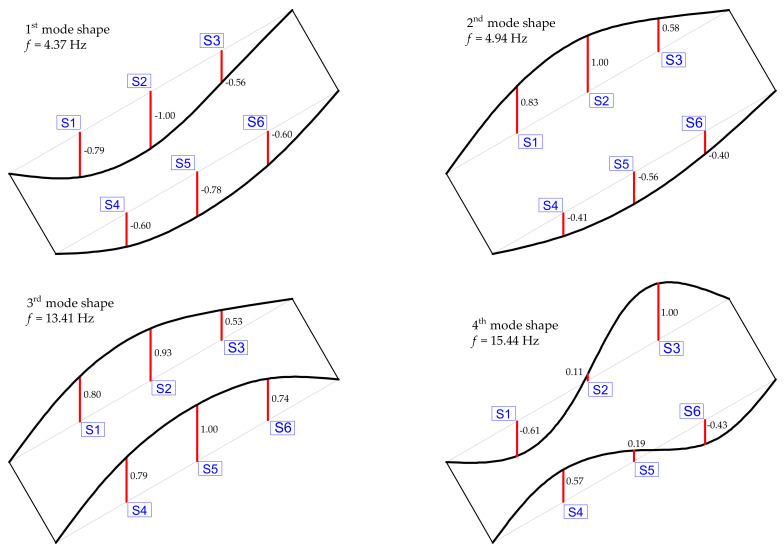
First four mode shapes identified from OMA before static load tests.

The list of natural frequencies identified from OMA is reported in Table 1 for each excitation configuration considered in the experimental campaign, while the first four mode shapes are illustrated in Figure 9. These results are relevant to the dynamic tests performed in the roadway from Palermo to Messina; however, similar results (not shown here for brevity) are obtained in the twin roadway from Messina to Palermo. It is worth noting that the mode shapes are identified by the modal coordinate corresponding to each of the six accelerometers. Since these six accelerometers are placed on the two sides of the bridge deck, it is not easy to distinguish between longitudinal flexural modes (mainly involving longitudinal PC girders) and transversal flexural mode (mainly involving transverse diaphragms) of the bridge deck. To capture this detail, additional accelerometers along the width of the deck (e.g., at W/4, W/2 and 3/4 W) should have been placed. Despite the relatively low number of signals, it is possible to foresee, based on the results in Figure 9, that the first and third vibration modes are of flexural type, while the second and fourth modes are of torsional type. These conclusions can be inferred by the critical analysis of the modal displacements: in the first and third modes the modal displacements related to the two sensor lines (i.e., S1-S2-S3 and S4-S5-S6) have the same sign, which indicates a flexural (longitudinal or transversal) deformation of the bridge deck, whereas in the second and fourth mode, they have opposite signs, which denotes a torsional deformation of the deck. The obtained results are useful for the development of the numerical FE model of the bridge deck, which is described in the following subsection.

**Figure 10:**
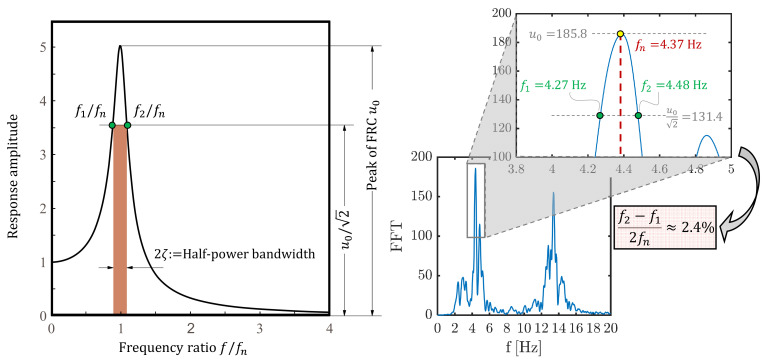
Identification of modal damping via half-power bandwidth: qualitative representation (**left**, adapted from Chopra [33]) and application to experimental frequency response curve for detecting the first mode damping ratio (**right**).

In addition to natural frequencies and mode shapes, dynamic tests are useful to identify the inherent damping ratio. Experimental measurements of damping ratios, typically achieved through the logarithmic decrement method or through the half-power bandwidth method [33], are useful to detect signs of damage of an existing structure. In this work, the second method is used to identify the damping ratio corresponding to the first and second modes of vibration. To this aim, the peak of the response amplitude (i.e., the resonant amplitude) $u_{0}$ is first identified from the frequency response curve (FRC), then the half-power bandwidth is computed from the frequency points at which the amplitude is $u_{0}/\sqrt{2}$ on either side of the resonant frequency $f_{n}$, namely $f_{1}$ and $f_{2}$. These two frequencies define an interval of frequencies, called half-power bandwidth, that is twice the damping ratio ζ, see Figure 10. Thus, the damping ratio can be estimated as:

(2) $$\frac{f_{2}-f_{1}}{f_{n}}=2\zeta .$$

The right part of Figure 10 shows the application of Equation (2) for the determination of the first-mode damping ratio based on the acceleration frequency response curves of the S1 signal, which results in $\zeta_{1}\approx 2.4\%$. Similar results are obtained for the other five signals. Experimental damping ratios (average values out of the six measures from the six accelerometers) of the first two modes identified for the two roadways separately are listed in Table 2. It is noted that average values of the first two damping ratios are approximately 2.5%, which are reasonable values for PC structural elements in elastic regime. These results suggest that there is no irreversible damage occurred or ongoing in the bridge deck, at least in the considered operating conditions.

### 4.2. Development and Preliminary Calibration of the Numerical FE Model

A simplified numerical FE model of the bridge deck is realized via a mesh of 1D beam elements representing the longitudinal PC girders and the transverse diaphragms, each incorporating a collaborating portion of RC slab whose effective width $b_{eff}$ is calculated according to prescriptions from NTC2018 [10] (in a similar fashion to what reported in Eurocode 4 for composite steel-concrete structures [34]). 

**Figure 11:**
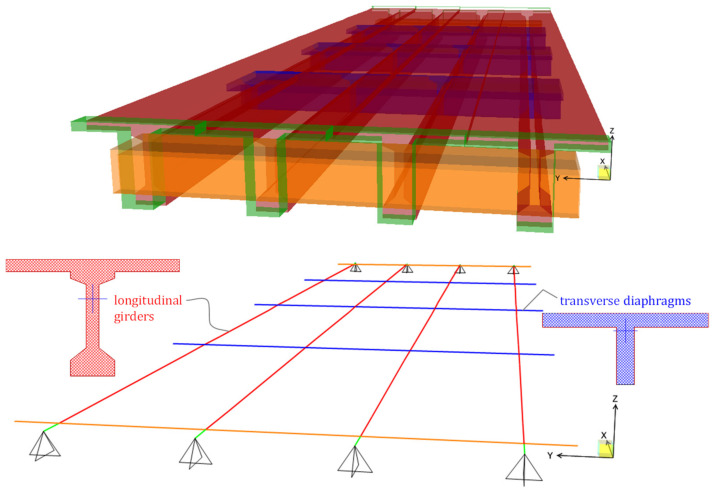
FE model of the bridge deck with 1D beam elements: extruded and standard view.

A sketch of the FE model realized with the structural analysis software SAP2000 [35] is illustrated in Figure 11. Only one span (length 29.00 m) is simulated in the model to reproduce the structural behavior of the bridge deck. Each node has six degrees of freedom. Pinned restraints are assumed at the abutment and at the intermediate pier. The exact geometry of the bridge deck is reproduced by also accounting for variation (widening) of the cross section at the two terminals of the girders, near the supports. Sectional properties (moments of inertia, torsional stiffness, etc.) are directly calculated by the Section Designer tool [35] that is integrated within SAP2000, based on geometrical input characteristics. A diaphragm constraint is assigned to all nodes of the bridge deck to simulate the membrane stiffening effect provided by RC slab and transverse diaphragms. Mass distribution is computed from the load analysis of the bridge deck, including self-weight of structural elements and superimposed dead loads (road pavement and guardrails).

**Figure 12:**
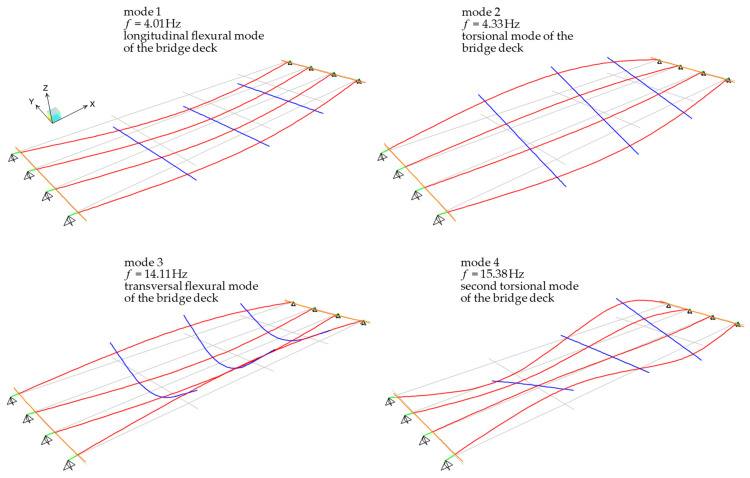
First four mode shapes obtained from FE model of the bridge deck.

Modal analysis (eigenvector analysis) is performed to determine the natural frequencies and mode shapes of the bridge deck. The first four modes of vibration along with corresponding frequencies are depicted in Figure 12. It is observed that the first (dominant) mode of vibration is, as reasonably expected, relevant to a longitudinal flexural mode of the bridge deck and has a frequency equal to 4.01 Hz. This frequency is only slightly different (8% lower) from the first natural frequency identified from OMA (4.37 Hz). The first natural frequency obtained by the FE model matches well with the theoretical first mode (flexural) frequency of a simply supported continuous beam with length L=29.00 m and cross-sectional properties of the PC longitudinal girders (i.e., area $A=1.13\;m^{2}$ and moment of inertia $J=0.4836\;m^{4}$). This first frequency can be analytically calculated as follows:

(3) $$f_{analytical}=\frac{\pi }{2}\sqrt{\frac{EJ}{\mu L^{4}}}=4.01\;\mathrm{Hz}$$

where E=34.660 GPa is the elastic modulus for concrete grade C35/45 (calculated in accordance with Eurocode 2 [36] expressions based on hypothetical 28-day concrete characteristic compressive strength equal to $f_{ck}=37.5\;\mathrm{MPa}$) and $\mu =G_{tot}/g$ is the linear mass density calculated as the ratio between the total permanent load acting on a single PC girder $G_{tot}=35.65\;\mathrm{kN}/m$ (from load analysis) and the acceleration of gravity g. It is worth noting that the analytical frequency in Equation (3) is perfectly identical to the first frequency detected from the numerical FE model of the bridge deck, which is reasonable considering that this first mode is related to the flexural longitudinal response of the bridge deck, thus uniquely involving the flexural behavior of the PC longitudinal girders.

A stiffer behavior of the bridge deck is identified from OMA (first two frequencies equal to 4.37 Hz and 4.94 Hz) compared to that obtained from the numerical FE model (first two frequencies equal to 4.01 Hz and 4.33 Hz), which may be due to the stiffening effect of the RC slab in the actual configuration of the bridge that is only approximately included in the simplified FE model, or to slightly different support conditions related to possible wear in the neoprene bearings. Apart from these minor differences, the first four mode shapes from the FE model are qualitatively consistent with the experimental one, cf. Figure 12 with Figure 9, and the comparison between experimental and numerical natural frequencies is also reasonably acceptable (relative errors are listed in Table 3). It can be concluded that the simplified FE model developed is accurate enough to capture the dynamic characteristics of the bridge deck. Further validation of the model is performed in the next subsection to simulate static load tests.

### 4.3. Static Load Tests

Static load tests are performed on the Longano viaduct to investigate the bridge structural behavior when subjected to service load conditions. The aim of these tests is to verify whether the bridge remains in elastic phase (without any cracking and damage sign) under the maximum allowed serviceability loads prescribed by the NTC2018 [10], by checking that the residual deflection measured after the load tests does not exceed 15% of the maximum deflection measured during the tests [10]. Code-conforming traffic loads on the bridge are computed based on the NTC2018 prescriptions [10] by dividing the deck in three equivalent lanes and applying uniformly distributed loads combined with tandem concentrated loads simulating heavy vehicle wheels. The worst loading position in longitudinal direction is first identified via girder analysis, and subsequently deck analysis is performed to evaluate the transversal distribution of loads.

For preliminary design of static load tests, a complete finite element model (FEM) is realized. Load schemes adopted for the static load tests are then designed to reproduce the same stress levels on the bridge deck (bending moments and shear forces) as those obtained under the characteristic combination of loads (with unitary combination coefficient for variable actions) prescribed by the NTC2018 [10].

**Figure 13:**
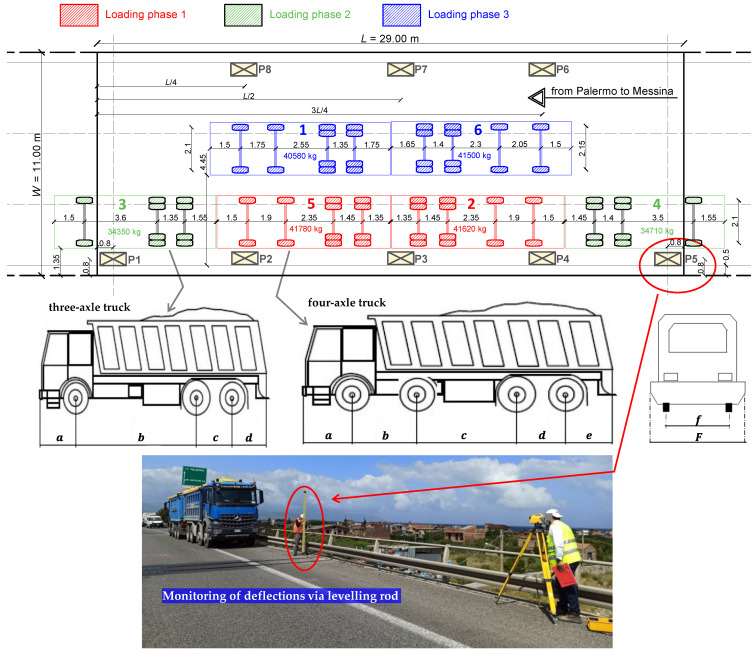
Plan of static load tests on the Longano viaduct deck with location of eight measurement points of displacement (P1–P8) and actual arrangement of three-axle and four-axle heavy trucks in three subsequent loading phases.

In the static tests, the loads are applied to the bridge deck by means of six heavy trucks identified by a unique ID (from 1 to 6), having either three or four axles. The truck loads are applied gradually in subsequent phases of increasing magnitude to check any possible sign of cracking or other damage-related phenomena. The layout of the actual truck loads relevant to the three phases is depicted in Figure 13 for the representative roadway from Palermo to Messina. However, a similar layout of truck loads is applied to the other roadway in the opposite direction (from Messina to Palermo).

Some measurement points of displacement are located on the two sides of the deck. More specifically, measurements of deflections are carried out via surveyor’s levels located in eight measurement points of displacement P1-P8, namely near the two end supports (P1 and P5), at the midspan (P3, P7) and at the two quarters of the bridge span (P2, P4, P6, P8). The sensitivity of the considered instrumentation is of the order of two hundredths of mm, which represents a reasonable threshold considering the deflection values usually measured in bridges (including those reported in this experimental campaign).

**Figure 14:**
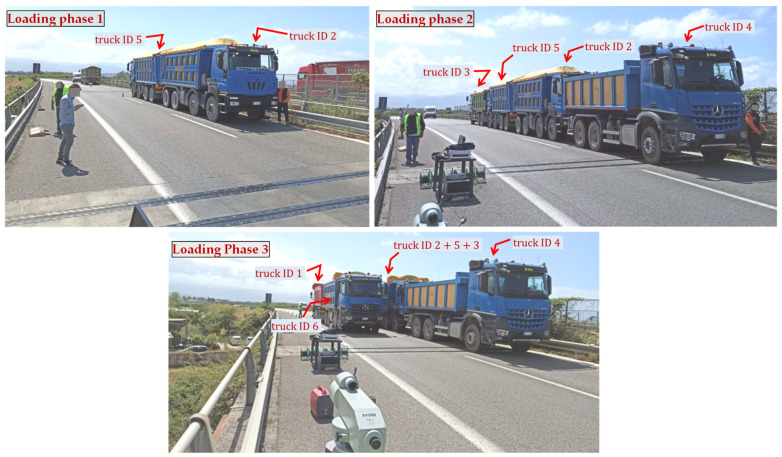
Photographs of static load tests in three subsequent phases for the roadway from Palermo to Messina.

Geometrical details of the trucks sketched in Figure 13 are listed in Table 4. Each truck is filled with coarse gravel such that the final gross mass reaches up to more than 34 t and 40 t for the three-axle and four-axle trucks, respectively. Some photographs taken during the various loading phases of the tests (pertinent to the roadway from Palermo to Messina) are reported in Figure 14. Between subsequent loading phases, a certain waiting time is expected to allow the stabilization of the deflection value. The time of loading phase 1 and 2 is of around 10 min, while that of phase 3 is of around 30 min to ensure stabilization of deflection. During the loading phases, the deck is monitored from below with a movable basket to control that all girders (including prestressing strands) do no exhibit any damage sign. After the three load steps, the unloading phase is performed by removing the truck loads following the opposite order of the loading phases.

**Figure 15:**
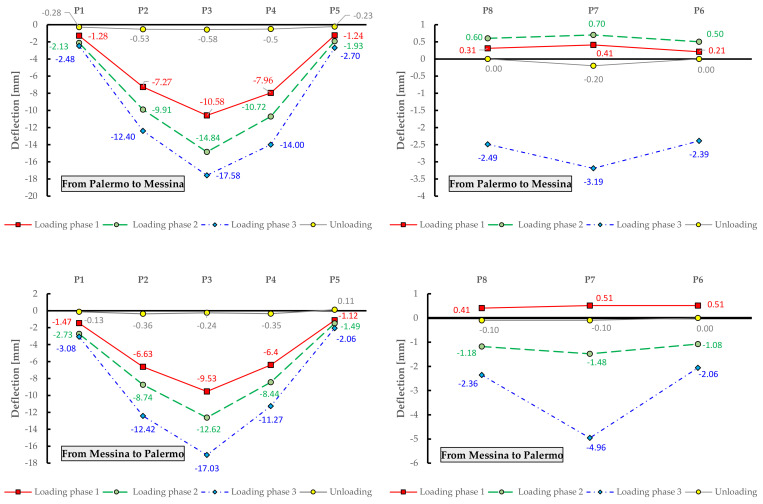
Experimental deflections for each measurement point obtained from static load tests on Longano bridge deck.

The measured deflections on the eight measurement points are reported in Figure 15, for each loading phase and for each traffic direction (roadway). It can be noted that the trend of the static deflections in the two roadways is substantially similar. Minor differences are observed when comparing the results from phase 2, which may be due to a slightly different arrangement of the trucks in the two roadways. The first loadings phases (especially phase 1) cause a positive displacement (i.e., raising of the deck) in the side opposite to the loading area (see measured deflections for points P6–P8) as the eccentric truck loads induce a torsional response of the bridge deck. The maximum deflections measured in the midspan of the deck (P3) are approximately 17 mm for both the roadways. It can be observed that the residual deflection measured upon unloading are negligible and are largely below the 15% limit threshold prescribed by the NTC2018 [10]. These results lead to the conclusion that the corrosion of the prestressing strands (documented above in Figure 3) does not affect the structural behavior of the bridge under service loads, i.e., does not produce decompression phenomena under service loads. This seems to reasonably indicate that the level of initial prestressing force that was considered in the design stage is large enough to compensate the reduction of resistant area of the prestressing strands induced by corrosion.

To support the above conclusions, additional dynamic tests are performed after the removal of truck loads to check whether the natural frequencies of the bridge deck are altered by the static load tests. Pertinent OMA results for the same excitation configurations described before (i.e., hammer pulse and truck load step) are listed in Table 5 for the Palermo to Messina roadway. Comparing the natural frequencies before (Table 1) and after (Table 5) the static load tests, minimal differences are found, which cannot be certainly ascribed to a potential accumulation of damage of the Longano viaduct. It can be inferred that the bridge deck remains in elastic regime under the maximum allowed serviceability loads prescribed by the NTC2018 [10].

### 4.4. Further Validation of the FE Model against Load Test Results

The numerical FE model of the bridge deck developed for simulating the dynamic tests is further validated against the load test results. To reproduce the actual load conditions of the tests, the FE model include self-weight of the structure, superimposed dead loads (road surface and parapets) and truck loads with appropriate distributions. For computational simplicity, it is assumed that the gross weight of each truck is equally distributed on the wheels.

**Figure 16:**
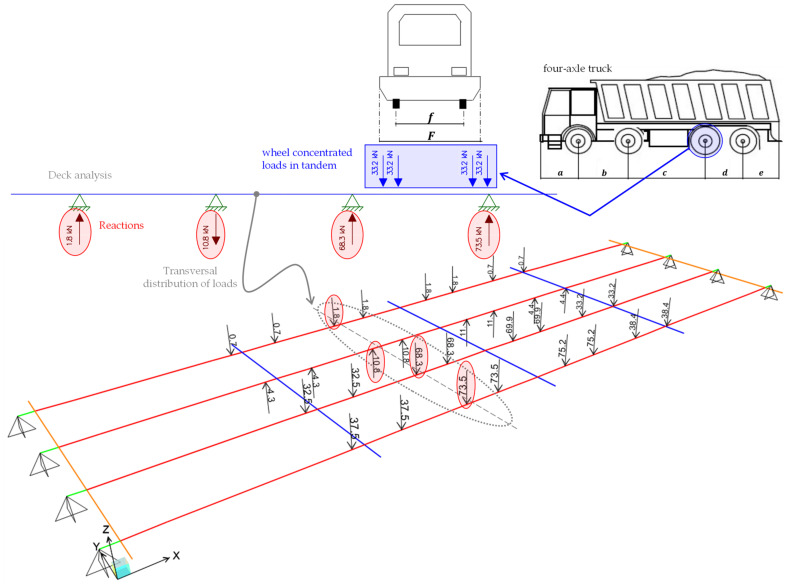
Simplified calculation of load distribution in FE model, representative example simulating loading phase 1.

Using 1D beams elements, loads can only be applied to the beam axis, whereas truck loads are transferred on the RC slab through wheel imprints that are generally not aligned with the beam axis. Consequently, to calculate equivalent loads on the longitudinal girders generated by the trucks located on the bridge deck, a simplified scheme for a reasonable transversal distribution is adopted as shown in Figure 16. Here, the reactions of the supports in the deck analysis are applied as concentrated loads (in opposite direction) to the longitudinal girders of the FE model.

**Figure 17:**
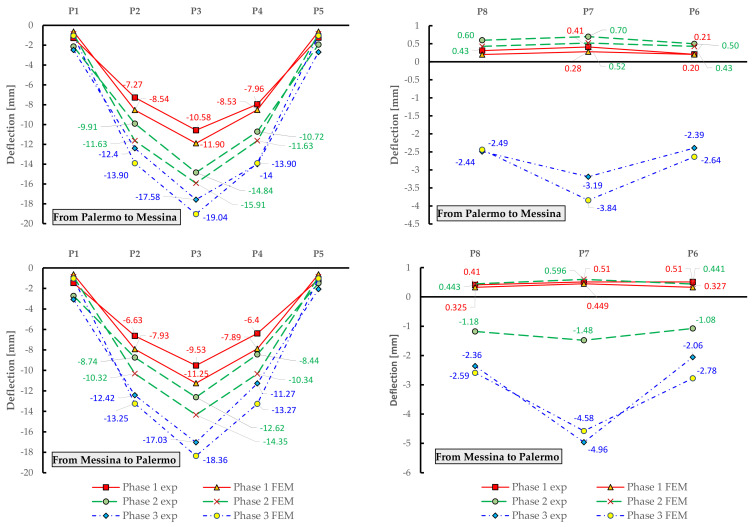
Comparison between experimental (exp) and numerical (FEM) displacements of the Longano bridge deck.

Once the truck loads are defined and implemented in the FE model, static linear analysis is performed to compute the deflections of the bridge deck. For comparative purposes, particular attention is paid to the deflections of those nodes closest to the eight locations of the measurement points considered in the experimental campaign. The comparison of the experimental (exp) and numerical (FEM) deflections is illustrated in Figure 17 for both the roadways (from Palermo to Messina and vice versa) and for the eight monitored points.

There is a reasonable agreement between the FE results and the experimental displacements, with average relative errors in the order of 10–15%. The highest discrepancies (30–40%) are observed in the measurement line P6-P7-P8, which is opposite to the loaded portion of the bridge deck. This may be ascribed to the simplified transversal load distribution adopted. It is also found that the FE model generally provides slightly larger deflections than the static load tests, which can be justified by the additional stiffening contribution of the RC slab that is only approximately incorporated in the resisting section of the longitudinal girders. Finally, some deviations between numerical and experimental results are noted (although not clearly shown in Figure 17) near the supports: it is expected that some minor displacements occur due to deformability of abutment and intermediate pier in the experimental case that are not captured in the FE model using pinned restraints that prevent translations in the three directions. Apart from these critical considerations, it can be concluded the analysis results further validate the numerical FE model. In the next section, the validated FE model is used to investigate the ultimate limit state behavior of the bridge deck accounting for different corrosion scenarios.

## 5. Nonlinear Static Analysis Accounting for Different Corrosion Scenarios

Based on the visual inspection, the bridge is subject to widespread corrosion phenomena concentrated only in the prestressing strands (cf. again Figure 3). Although it has been experimentally and numerically demonstrated that the corrosion does not affect the structural behavior of the bridge under service loads, it is reasonable to expect that the load-bearing capacity of the bridge deck at the ultimate limit states is seriously influenced by the corrosion-induced reduction of the cross-sectional area of the prestressing strands. Unfortunately, no experimental measurements of the actual corrosion rates are available so far, but they are planned for the near future. However, it is of utmost importance to gain insights, or at least to obtain preliminary estimates, on how the corrosion of steel strands affects the load-bearing capacity of the bridge. To this aim, this section is devoted to investigating, from a numerical point of view, the ultimate limit-state behavior of the bridge deck accounting for different (hypothetical) corrosion scenarios of prestressing strands. The results from this numerical investigation will be useful to quantify the risk level of the infrastructure associated with different extents of corrosion (whose actual values will be identified through subsequent experimental findings).

Numerical static nonlinear analysis under incremental loads is performed to quantify the post-elastic structural response focusing on the reduction of the load-bearing capacity of the bridge deck induced by corrosion. It is worth noting that in the literature many models were developed to describe the effect of corrosion on the strength and ductility of RC structures [37,38]. Generally, the reduction of mechanical properties is more severe for pitting corrosion than for uniform corrosion, especially in presence of permanent cracking. Without any experimental results concerning the actual corrosion state of the bridge deck and considering the absence of cracking, here, some simplified hypotheses must be declared; in particular, a status of uniform corrosion of the prestressing strands is assumed and the indirect degradation of the mechanical properties of surrounding concrete close to corroded steel is neglected. For comparative purposes, six different nonlinear FE models of the bridge deck are realized, each characterized by a different corrosion rate (CR), or mass loss in percentage, namely 0% (uncorroded bridge configuration), 5%, 10%, 20%, and 30%. In each FE model, the distribution of vertical loads is consistent with the static load tests. Assuming as initial condition the scheme of the loading phase 3 (cf. Figure 13), these loads are increased monotonically up to the collapse of the structure. The choice of this initial loading configuration (loading phase 3 related to the static load tests previously described) is motivated by the eccentricity of the loads in the deck that generate simultaneous flexural effects on the girders and torsional effects of the deck.

To perform nonlinear static analysis, material nonlinearity is incorporated in the numerical FE model. A concentrated plasticity approach based on plastic hinges located in some specific portions of the beam elements is adopted. Preliminary calibration of the moment-curvature M−χ relationships of some representative sections of the longitudinal girders, namely section A-A, B-B and C-C depicted in Figure 2b), is made. These three sections are characterized by three different positions of the prestressing strands and, thus, by different yielding and ultimate moment values and moment-curvature relationships. Assuming an ultimate tensile strength of the prestressing strands of longitudinal girders equal to $f_{pk}=1700\;\mathrm{MPa}$, the resulting characteristic yielding stress is $f_{p\left(0.1\right)k}=1544\;\mathrm{MPa}$ (a hardening ratio equal to 1.1 is adopted). Initial stress (allowable stress) is then calculated as $\sigma_{pi}=0.90f_{p\left(0.1\right)k}=1360\;\mathrm{MPa}$ according to EC2 [39,40]. Since the Longano viaduct was built in 1970, stress losses in the prestressing strands play an important role in the definition of the sectional capacity and cannot be ignored in the analysis. The stress losses due to concrete shrinkage, creep, and steel relaxation are computed and combined in accordance with Eurocode 2 expressions [39], assuming class 1 for ordinary wires and strands [40]. The final value of combined stress losses is equal to 345.45 MPa, thus leading to an effective stress for the strands equal to 1016.55 MPa. This value is used to compute the M−χ relationships for the calibration of the plastic hinges.

**Figure 18:**
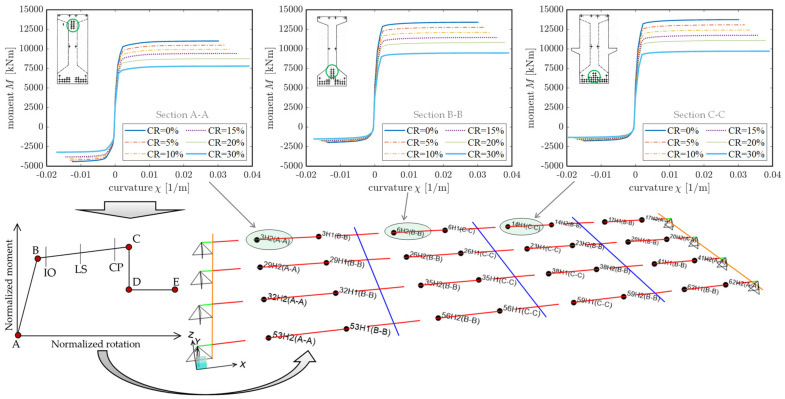
FE model of the corroded bridge deck with material nonlinearity simulated through a set of plastic hinges in longitudinal girders (concentrated plasticity approach) with properly calibrated moment-curvature relationships.

The nonlinear (concentrated-plasticity) FE model of the bridge deck is shown in Figure 18 along with the corresponding moment-curvature laws for the three representative sections A-A, B-B, and C-C, and for the six corrosion scenarios analyzed in this study. It can be observed that the ultimate moment decreases with increasing values of CR, while the ultimate curvature increases with CR because the failure tends to be more ductile, as it is achieved with a higher strain level in the corroded prestressing strands (having reduced resistant sections due to corrosion) than the uncorroded case. From the M−χ relationships, normalized moment-rotation curves are constructed and implemented in SAP2000 [35] based on a plastic hinge length equal to the section height. In a separate model, plastic hinges are also calibrated and implemented for the transverse diaphragms, besides those in longitudinal PC girders; however, it has been found that the yielding moment of these transversal elements, even in the most extreme loading and corrosion scenarios, is never exceeded, which implies that transverse diaphragms can be assumed as linear elastic elements for computational simplicity.

**Figure 19:**
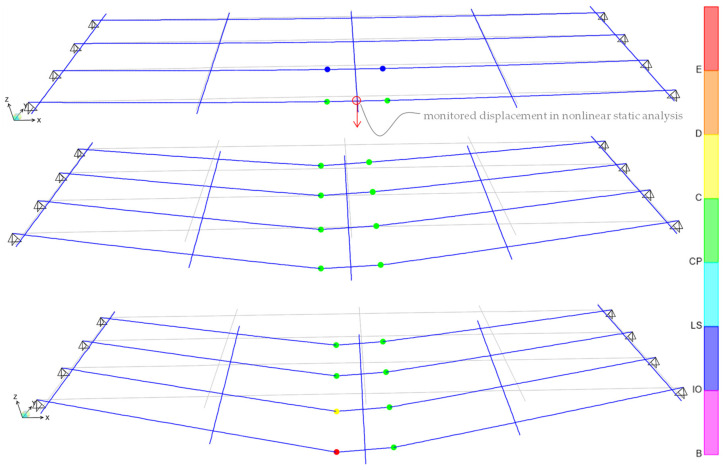
Deformed shape and plastic hinge development corresponding to three incremental loading steps (of increasing magnitude) during the nonlinear static analysis.

Displacement-controlled nonlinear static analysis is carried out in SAP2000 [35], by monitoring the displacement of the midspan node (on the roadway side that is mostly loaded) under loads of increasing amplitudes, as sketched in Figure 19. As reasonably expected, the development of the plastic hinges under monotonically increasing loads indicates that the most critical sections are those located near the mid span.

**Figure 20:**
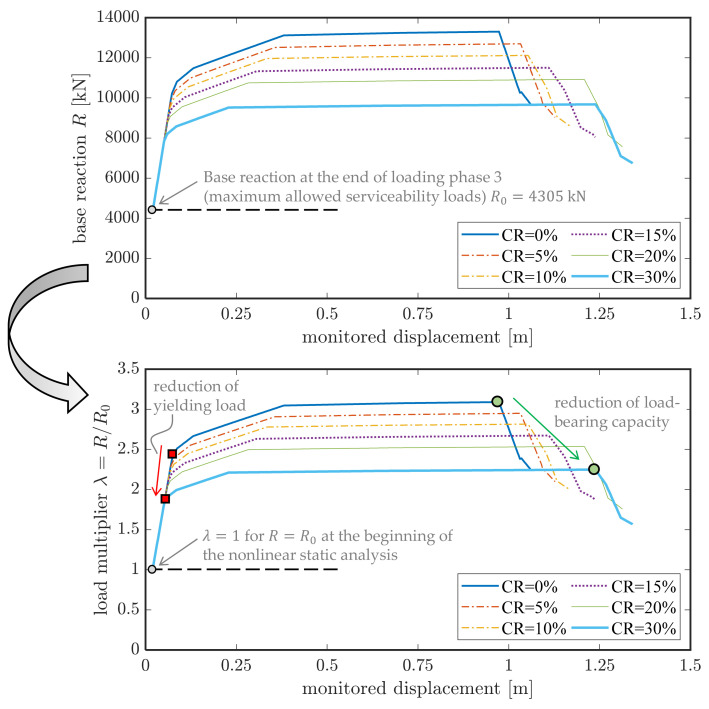
Results from nonlinear static analysis of the bridge deck: pushover curve (**top**) and corresponding load multiplier λ computed by normalizing the base reaction with respect to the value obtained at the end of loading phase 3 (**bottom**).

The pushover curves in terms of base reactions (sum of reactions of abutment supports and intermediate pier supports) versus monitored displacement are shown in Figure 20. It can be observed that the pushover curve starts from a base reaction $R_{0}=4305\;\mathrm{kN}$ for all the corrosion scenarios, which corresponds to the end of loading phase 3, i.e., the reaction computed under the maximum allowed serviceability loads. Further load increase (up to yielding load 8000–10,000 kN, depending on CR) is allowed by the bridge deck while remaining in elastic regime, after which the behavior becomes nonlinear. The corrosion evidently affects (reduces) the load-bearing capacity of the bridge deck.

For the generic step s of the nonlinear static analysis, the base reaction can be expressed as $R\left(s\right)\;=\;\lambda \left(s\right)\cdot R_{0}$, where $\lambda \left(s\right)$ represents a dimensionless load multiplier at the considered step s that quantifies the increase of the load beyond the elastic threshold at the end of loading phase 3. In addition to the pushover curve in dimensional form, the bottom part of Figure 20 reports the trend of such load multiplier λ versus the monitored displacement for the six considered corrosion scenarios. It can be easily observed that both the yielding load (i.e., the base reaction corresponding to the formation of the first plastic hinge) and the load-bearing capacity of the bridge deck (peak of the pushover curve) decrease with increasing CR values.

**Figure 21:**
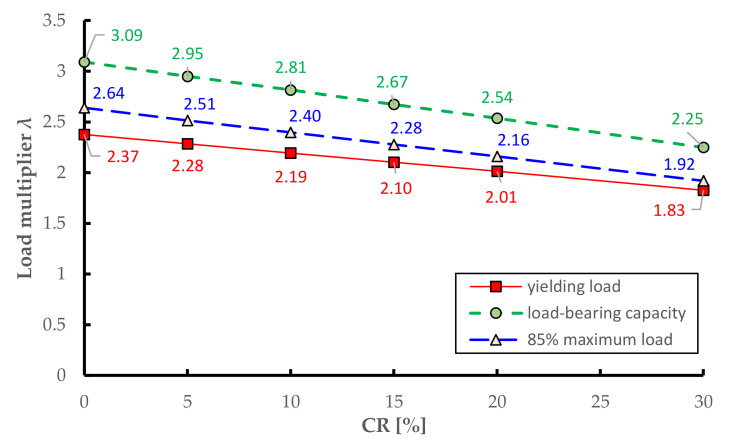
Reduction of load multipliers obtained from nonlinear static analysis of the bridge deck for increasing corrosion scenarios.

To summarize the main results, Figure 21 illustrates the trend of three characteristic load multiplies versus the CR value, namely the yielding load, the load-bearing capacity (maximum load) and the ultimate load calculated as the 85% of the maximum load. These diagrams are useful to preliminarily estimate the reduction of the load-bearing capacity of the bridge deck depending on the corrosion rate. The yielding load multiplier in the uncorroded case is 2.37, which means that the first plastic hinge formation occurs at a load level approximately two times and a half higher than that simulated in the loading phase 3 for the on-site static tests. This is a relatively high value considering the time of construction of the bridge, which confirms that the bridge is rather safe under the service loads prescribed by the current regulations NTC2018 [10]. Moreover, the corrosion leads to a considerable drop of the three load multipliers, following a linear trend with almost comparable slope. In particular, the following linear regression formula well describes the decreasing trend of the load-bearing capacity of the bridge deck:

(4) $$F_{\mathrm{corroded}}=F_{\mathrm{uncorroded}}-0.028\cdot \mathrm{CR}\left[\%\right]$$

which is valid under the simplified assumption of uniform corrosion for all the prestressing strands and within the limitations of corrosion levels considered in this numerical study (i.e., up to 30%). This formula can be applied to predict a first estimate of the corrosion-induced reduction of the load-bearing capacity of existing PC bridge decks sharing similar features to the Longano viaduct here analyzed as case study. This is particularly important when a large infrastructure network is analyzed, in which generally many bridges are similar from the perspective of (aggressive) environmental conditions, static scheme and age of construction. Therefore, it is expected that the degradation of material parameters is comparable for a series of bridge structures belonging to the same infrastructure network. As an example, for a reasonable corrosion rate equal to 10%, Equation (4) predicts a considerable reduction of the load-bearing capacity of almost 30%. Although this is only a preliminary estimate that is acceptable under the strict assumption of uniform corrosion, in most cases it is difficult to obtain a punctual and precise evaluation of the mass loss in percentage of each prestressing strand. Indeed, many practical difficulties exist when performing experimental measurements, including (among others) costs and feasibility considerations for finding a convenient access to girders and other structural elements. Considering such circumstances, the proposed experimental-numerical approach can be used as a preliminary assessment tool to identify critical parts of a large infrastructure network prior to performing widespread and expensive material test campaigns.

## 6. Conclusions

This paper has presented a systematic approach for the preliminary assessment of the load-bearing capacity of existing PC bridge decks by combining experimental measurements and numerical analyses. The methodology proves to be particularly useful for existing PC bridges exhibiting corrosion in the prestressing strands in order to ascertain whether and to what extent these phenomena affect the structural behavior of the bridge in both serviceability and ultimate conditions. The main steps of the procedure, here applied to the real case study of the PC bridge deck of the Longano viaduct (southern Italy), are summarized as follows:

1. Dynamic identification tests are preliminary performed based on OMA from vibration data collected in situ. These tests are performed in free vibration mode and do not cause any damage to the bridge deck. In addition to providing natural frequencies and mode shapes of the bridge deck, these measurements also serve to obtain estimates of the damping ratios (for instance, by the half-power bandwidth method), which are useful indicators of the potential ongoing damage of the bridge deck.
2. The results of the dynamic tests from step 1 are then used to develop and calibrate a numerical FE model. For simplified estimates of the structural response, 1D beam elements can be used for the structural analysis of the bridge deck.
3. Static load tests are performed in situ to investigate the structural behavior (deflections) of the bridge deck under service loads. These tests are designed and performed in different loading phases of increasing amplitude to avoid sudden damage of structural members in case of advanced material deterioration state. The test results are used to study whether the bridge behavior remains in elastic regime under the maximum allowed (code-conforming) serviceability loads, i.e., by checking that the residual deformation upon unloading does not exceed a critical threshold, which may be an indicator of irreversible damage in some part of the bridge. These test results, if possible, should be followed by a second series of dynamic tests to compare the natural frequencies after the application of loads with those identified in the previous step 1.
4. The results of the static load tests from step 3 are then used to further verify the validity of the FE model developed in step 2, by reproducing the actual load conditions of the tests and comparing numerical deflections with experimental measurements.
5. In addition to the response under service loads, numerical static nonlinear analysis with the previously validated FE model is performed to investigate the influence of different corrosion scenarios on the resulting structural behavior of the bridge deck at ultimate limit states. This numerical analysis is useful to quantify the variation of the load-bearing capacity depending on some hypothetical corrosion rates of the prestressing strands. To this aim, a regression formula has been proposed based on the numerical results found in this study that can be utilized to obtain preliminary estimates of the corrosion-induced degradation of the bridge structural performance for other viaducts sharing similar features to those of the Longano viaduct here analyzed as case study.

In the authors’ opinion, the proposed experimental-numerical framework can provide a rapid overview of the bridge structural health in both serviceability and ultimate conditions without performing detailed and extensive tests on material and structural components (which would be certainly needed for deepening the level of investigation). In this context, the methodology can represent a convenient assessment tool to rapidly identify critical portions of a large infrastructure network prior to performing detailed analyses to establish a list of intervention priorities in a timely and reasonable way. Future research developments concern the validation of the findings from this study with additional material test results, and the comparison of the numerical outcomes with alternative time-dependent approaches available in the literature.

## Figures and Tables

**Table 1:** Identified natural frequencies from OMA related to different excitation configurations before static load tests.

Excitation Configuration	$f_{1}\;\left[Hz\right]$	$f_{2}\;\left[Hz\right]$	$f_{3}\;\left[Hz\right]$	$f_{4}\;\left[Hz\right]$
hammer pulse load at midspan	4.45	4.88	13.48	-
hammer pulse load at a quarter of span	4.39	4.88	13.43	15.44
truck load step at midspan	4.50	4.87	13.18	-
truck load step at a quarter of span	4.37	4.94	13.41	-

**Table 2:** Experimental damping ratios (average values) for the first two modes of vibration.

Mode Shape	Average Damping Ratio ζ Messina to Palermo Roadway	Average Damping Ratio ζ Palermo to Messina Roadway
mode 1	2.51%	2.10%
mode 2	2.39%	2.61%

**Table 3:** Comparison between experimental and numerical natural frequencies of the bridge deck.

Mode Number	Type of Vibration (Exp = FE Model)	Frequency (Exp) [Hz]	Frequency (FE Model) [Hz]	Relative Error [%]
1	longitudinal flexural	4.37	4.01	8.23
2	1st torsional	4.94	4.33	12.35
3	transversal flexural	13.41	14.11	5.22
4	2nd torsional	15.44	15.41	0.19

**Table 4:** Characteristics of the heavy trucks used for the load tests on the Longano viaduct.

Truck ID	N. of Axles	$a\;\left[m\right]$	$b\;\left[m\right]$	$c\;\left[m\right]$	$d\;\left[m\right]$	$e\;\left[m\right]$	$f\;\left[m\right]$	$F\;\left[m\right]$	Gross Mass [kg]
1	4	1.50	1.75	2.55	1.35	1.75	2.10	2.60	40,580
2	4	1.50	1.90	2.35	1.45	1.35	2.10	2.60	41,620
3	3	1.50	3.60	1.35	1.55	-	2.10	2.60	34,350
4	3	1.55	3.50	1.40	1.45	-	2.10	2.60	34,710
5	4	1.50	1.90	2.35	1.45	1.35	2.10	2.60	41,780
6	4	1.50	2.05	2.30	1.40	1.65	2.15	2.65	41,500

**Table 5:** List of identified natural frequencies from OMA after static load tests (to compare with results from Table 1).

**Excitation Configuration**	$f_{1}\;\left[Hz\right]$	$f_{2}\;\left[Hz\right]$	$f_{3}\;\left[Hz\right]$	$f_{4}\;\left[Hz\right]$
hammer pulse load at midspan	4.39	4.87	13.48	-
hammer pulse load at a quarter of span	4.41	4.82	13.43	15.38
truck load step at midspan	4.34	4.85	-	-
truck load step at a quarter of span	4.37	4.88	13.42	-

## Data Availability

Data sharing not applicable.
